# Fixed Versus Rotating Practice Monitors in Small-Group Anatomy Teaching: A Prospective Study in Medicine and Optometry

**DOI:** 10.1007/s40670-026-02793-6

**Published:** 2026-06-29

**Authors:** María Carmen Garza, Irene Gracia-Rubio, María José Luesma, Isabel Lamiquiz, Manuel Lahoz, Itziar Lamiquiz-Moneo

**Affiliations:** 1 Department of Human Anatomy and Histology, School Medicine, University of Zaragoza, Zaragoza, Spain; 2 Unidad de Lípidos, IIS Aragón, CIBERCV, Hospital Universitario Miguel Servet, Avda. Isabel La Católica 1-3, Zaragoza, Zaragoza 50009 Spain; 3 Department of Psychology and Sociology, Faculty of Social and Labor Sciences, University of Zaragoza, Zaragoza, Spain

**Keywords:** Practice system, Fixed practice monitor, Rotating practice monitor, Anatomy, Medicine, Optometry

## Abstract

**Introduction:**

Small-group practice sessions facilitated by peer or faculty monitors are widely used in health professions education to support the integration of theoretical knowledge. However, evidence regarding the optimal organizational model for assigning practice monitors is limited. This study compared fixed versus rotating practice monitor systems in terms of knowledge acquisition and student satisfaction.

**Methods:**

A prospective experimental study was conducted involving 198 medical students and 58 optometry students. Medical students were allocated to either a fixed or rotating practice monitor system and completed three formative Kahoot! based quizzes at 6 weeks, 4 weeks, and 2 days prior to the final examination. Optometry students experienced both systems, using fixed monitors in the first half of the course and a rotating system in the second half. Student satisfaction was assessed using an online survey in both degree programs.

**Results:**

Medical students assigned to fixed practice monitors achieved significantly higher scores in the first two formative quizzes; however, no significant differences were observed in the final quiz or final examination results. Similarly, no significant differences in examination performance were found between practice monitor systems among optometry students. Student satisfaction ratings were comparable across both organizational models.

**Conclusions:**

Fixed practice monitor systems may support more consistent early learning progress, but overall knowledge acquisition and academic performance did not differ between fixed and rotating models. These findings suggest that organizational flexibility in assigning practice monitors can be implemented without compromising learning outcomes, offering practical guidance for curriculum planning in health professions education.

**Supplementary Information:**

The online version contains supplementary material available at https://doi.org/10.1007/s40670-026-02793-6.

## Introduction

Anatomy is considered the basic of medical practice, particularly in surgical specialties, as well as in other non-medical specialties related to medicine, such as physiotherapy, nursing, optometry or occupational therapy, due to its direct relevance in daily clinical practice [1–4]. Despite the importance of anatomy, its teaching in university programs appears to be declining, evidence by reduced contact hours and concerns regarding ethical validity and the expenses of traditional cadaveric dissection [5–7].

Teaching methods in anatomy used to include didactic teaching, cadaveric dissection, the use of anatomical models, reconstructions by planes and coronal, sagittal and horizontal macroscopic Sect [8]. However, rapidly advancing technologies have undeniably altered these teaching anatomy methods with a recent shift towards game-based learning, computer-assisted learning, and simulation tools such as virtual reality glasses [9, 10]. Numerous studies have investigated the effectiveness of these modern teaching methods, which have proven very useful as supplementary tools in enhancing students attention and interest [3, 11]. Emphasis has also been placed on student learning, based on their characteristics and personality types, considering new styles and learning approaches [12].

Despite all the advances, cadaveric dissection is considered a traditional technique that is difficult to replace providing unique learning experiences that cannot be replicated with technology-based learning alone [13, 14]. This traditional teaching method, applied at the most of universities, has been the primary anatomy teaching method for over 400 years [15, 16]. The cadaveric dissection is conducted with small group of students (15–20 students) seated around the anatomical tables with an explanation of the parts by the teacher, and soon after the demonstration, the class is finished [14]. Similar to dissection, the utilization of anatomical models and natural specimens is more effective in enhancing the quality of practical lessons, particularly when conducted in smaller group [17–19]. The problem arises when the groups are much larger than 20 students. To solve this problem, the methodology applied in the Anatomy lessons in Medicine and Optometry Degree at the University of Zaragoza consists of dividing the students into groups of 5 to 8 students, in which there is a practice monitor who is in charge of directing the practice. This practice monitor, who has previously received the practical lesson from the teacher, will be in charge of guiding the practice for the rest of his classmates. Traditionally, practice monitors were chosen from the top-performing students, who demonstrated a strong interest and had the highest previous grades. However, in recent years, with the aim of providing students with more equitable and inclusive opportunities and fostering greater involvement, the approach has shifted towards electing a rotating practice monitor. Despite the widespread use of peer-led practical sessions, evidence comparing different organizational models for assigning student monitors remains limited. Therefore, the objective of this study is to determine which of the two practice methods, fixed or rotating practice monitors, is most effective for knowledge acquisition among students of Medicine and Optometry, enrolled in Neuroanatomy and Human Anatomy and Histology, respectively, at the University of Zaragoza.

## Materials and Methods

### Study Design

A prospective experimental study was carried out on a sample of 198 students from Medicine grade, and a sample of 58 students enrolled in Optometry grade in the same class group. Medical students were second-year students enrolled in Neuroanatomy at the University of Zaragoza (Spain) during the 2022–2023 academic year. Optometry students were first-year students enrolled in Human Anatomy at the University of Zaragoza as well, but during the 2023–2024 academic year. The study protocol was approved by the local ethical committee institution (Ethics and Clinical Research Committee of Aragón); all procedures were in accordance with the ethical standards of that committee and with the principles embodied in the Declaration of Helsinki.

Both subjects, Neuroanatomy and Anatomy and Histology, are equivalent to 9 credits of the European Credit Transfer and Accumulation System (ECTS). The teaching method of both subjects is based on master classes along with practical sessions in the dissection room, with cadavers, plan anatomy atlases, brain sections stained with the Weigert method, anatomy mockup and natural specimens. Medical students receive 4–5 theoretical classes and 2 h of practice per week during the second semester of their second year of Medicine degree (Supplemental Table 1). Optometry students receive 3 theoretical classes and 1.5 h of practice per week throughout their first year in the Optometry degree (Supplemental Table 2).

The practical lessons are classes in which students autonomously review theoretical knowledge using models and natural specimens. Medical students are distributed into groups of 5 to 8 people for 16 practical lessons, and these groups remain fixed throughout the year. In contrast, Optometry students are divided into 5 fixed groups of 10 to 11 students each.

Each Monday for medical students, or at the beginning of each practical session, teachers give practical lessons to a small group comprising students who will play the role of monitor from each regular practical group. During these sessions, teachers specify the anatomical concepts to be addressed the subsequent week, building upon those previously presented in theoretical classes. Then students, will be guided in practical sessions by their monitors, in a peer-teaching experience. 

### Students

#### Medicine Grade

Regarding medical students, they are divided in two distinct class groups (Group A and B). Group A contains 105 students, and from those, 20 students were selected as fixed practice monitors at the beginning of the semester, based on achieving the highest marks in the previous anatomical subject, splanchnology. This number was chosen to ensure a substitute monitor was always available, despite there being 16 practical sections and these monitors remain consistent throughout the semester; hence, they are referred to as fixed monitors.

Unlike Group A, where practice monitors are fixed, in Group B, comprising 93 enrolled students, the role of practice monitor rotates among the students within each anatomical section. The designated practice monitor is responsible for guiding the practical lessons for one week. Consequently, each week, a different student from each anatomical section assumes the responsibility of being the practice monitor.

The final exam was the same for both groups and consisted of two different parts: (a) theory exam containing 30 multiple-choice questions, which represented 60% of final grade; (b) an image exam featuring various questions related to cadavers, anatomy atlases, and natural specimens, accounting for 30% of the final grade. The remaining 10% of the final grade derived from continuous evaluation, attending practices, seminars, as well as the preparation of neuroanatomy-related clinical cases. (Supplemental Figure). 

#### Optometry Grade

Regarding to Optometry students, during the first semester, students have fixed monitor practice (selecting the 5 best students) and during the second semester, students have rotating monitor practice. At the end of each semester a final exam is taken, which represented 50% of final grade and consisted in a theory exam with 30 multiple-choice questions and two theoretical subjects to be elaborated upon. The practical concepts, which account for 25% of the overall grade, are continuously assessed during the development of the practices. Students who do not pass the practical component through continuous assessment will be required to take a practical examination in the dissection room. The remaining 25% of the final grade is obtained from continuous evaluation, attending practices, seminars, preparation of flipped classroom exercises as well as the preparation of clinical cases (Supplemental Figure).

### Kahoot! Exercises

Three Kahoot! exercises were conducted throughout the entire semester in Medical students in both groups. The first Kahoot! exercise took place six weeks prior to the exam, the second four weeks before, and the third two days before the exam. The Kahoot! schedule correspond to the completion of each of the theory-practice blocks. The students, completed the unannounced Kahoot! exercise at the end of a practice session in groups, identifying themselves by their anatomical section number. (Supplemental Figure).

Considering the content which had to be evaluated in the courses, 10–11 multiple-choice questions were designed in Kahoot! Exercises. Each question had only one correct answer and a limited response time of 20 s. Before starting to use the educational game, the students were given instructions and the option to either download the application on their mobile devices or use it online.

### Perception of Practices

Perception of each type of practices methodology was collected by a Google Form survey administered to students in both Medicine and Optometry grades. This survey comprised nine questions utilizing Likert scales to assess the type of practice monitor they had experienced. It also inquired about which type of monitor – fiexed versus rotating – they considered best and asked for their opinions on the advantages and disadvantages of each monitor type. The survey link was sent by internal university email to each of the 198 students enrolled in the Medicine grade and 58 students enrolled in Optometry grade.

### Statistical Analysis

Continuous variables are expressed as mean ± SD or median (25th percentile − 75th percentile) as applicable and categorical (nominal) variables are reported as percentages of total sample. Differences between independent variables were calculated by T-Student or U Mann Whitney test, as appropriate, while categorical variables were compared using the chi-squared test. The evaluation of the acquisition of knowledge based on the type of bosses generated in (Fig. 2) was made with the ggplot2 package. All statistical analyses were performed with R version 3.5.0 and significance was set at *p* < 0.05. In addition to the satisfaction survey, the grades from all students were statistically analyzed in order to measure the effectiveness of the two types of practice monitors from both a qualitative and a quantitative perspective.

## Results

From the 105 students enrolled in the group A, which had fixed practice monitors, the 20 students selected as fixed practice monitors, as expected due to their higher grades in splanchnology, also achieved significantly higher marks in the final exam and in each of its components (image and theory exams) compared to the remaining students in Group A who were not selected as fixed practice monitors (*p* < 0.001 in all case, Table 1).

**Table 1:** Comparation in the overall exam between Medicine students who had elected as fixed practice monitors versus rest of Medicine students who had fixed practice monitors

	Medicine students who were elected as fixed practice monitors (N=20)	Medicine students who were not elected as fixed practice monitors (N=85)	p
Final grade	9.39 ± 0.45	7.75 ± 1.43	<0.001
Theory exam	9.35 ± 0.52	7.85 ± 1.37	<0.001
Image exam	9.47 ± 0.55	7.56 ± 1.92	<0.001

Quantitative variables were expressed as mean ± standard deviation. The p value was calculated by Wilcoxon test

**Fig. 1:**
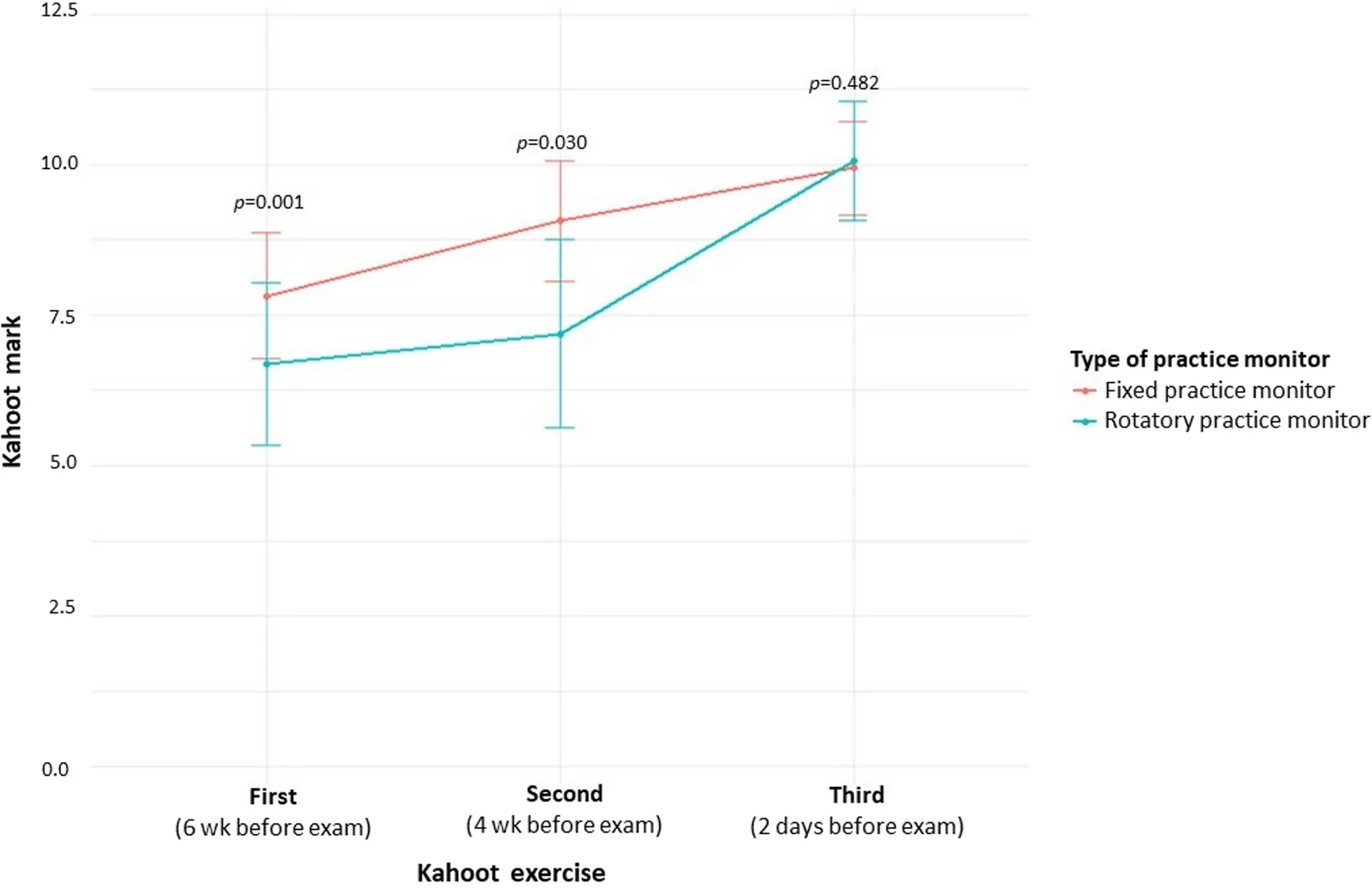
Evolution of Kahoot marks depending of the type of practice monitor system in Medical students. The *p* value was calculated by U the Mann Whitney test. wk denotes weeks

The evolution of Kahoot! scores, among Medical students, based on the type of practice monitor, was assessed, demonstrating a significant difference in knowledge acquisition throughout the course between the two groups (Fig. 1). Specifically, students with fixed practice monitors achieved significantly higher Kahoot! scores in the first and second exercises compared to those with rotating practice monitors (*p* = 0.001 and *p* = 0.030, respectively, Fig. 1). However, these differences diminish over time, and by the final Kahoot! Exercise, held just two days before the exam, there was no longer any difference between the two practice systems (*p* = 0.482, Fig. 1). It is important to note that there were not significant differences between both groups in their final marks for the previous anatomical subject, splanchnology (*p* = 0.154).

The overall exam marks for students enrolled in Neuroanatomy who had fixed practice monitors *versus* those who had rotating practice monitors is shown in Table 2. Although the knowledge acquisition of students showed significantly time-course differences depending on the type of practice monitor, the final marks were not significantly different between the two groups. In fact, we found that students with fixed practice monitors had analogous final grades (theory and imagen exam) that students compared to students with rotating practice monitors (*p* = 0.736, *p* = 0.792 and *p* = 0.090, respectively, Table 2).

**Table 2:** Comparation in the overall exam marks between Medicine students enrolled in Neuroanatomy subject 2022-23 who had fixed practice monitors versus Medicine students who had rotating practice monitors

	Medicine students who had fixed practice monitors (N= 105)	Medicine students who had rotating practice monitors (N= 93)	p
Final grade	8.07 ± 1.45	8.20 ± 1.38	0.736
Theory exam	8.14 ± 1.38	8.08 ± 1.41	0.792
Image exam	7.93 ± 1.90	8.42 ± 1.61	0.090

Quantitative variables were expressed as mean ± standard deviation. The p value was calculated by Wilcoxon test

From 58 students enrolled in Optometry grade, we found no significant differences between the final exam grades of each semester (*p* = 0.695). The average grade was 5.20 ± 3.08 compared to 5.00 ± 3.14 during the second semester.

**Fig. 2:**
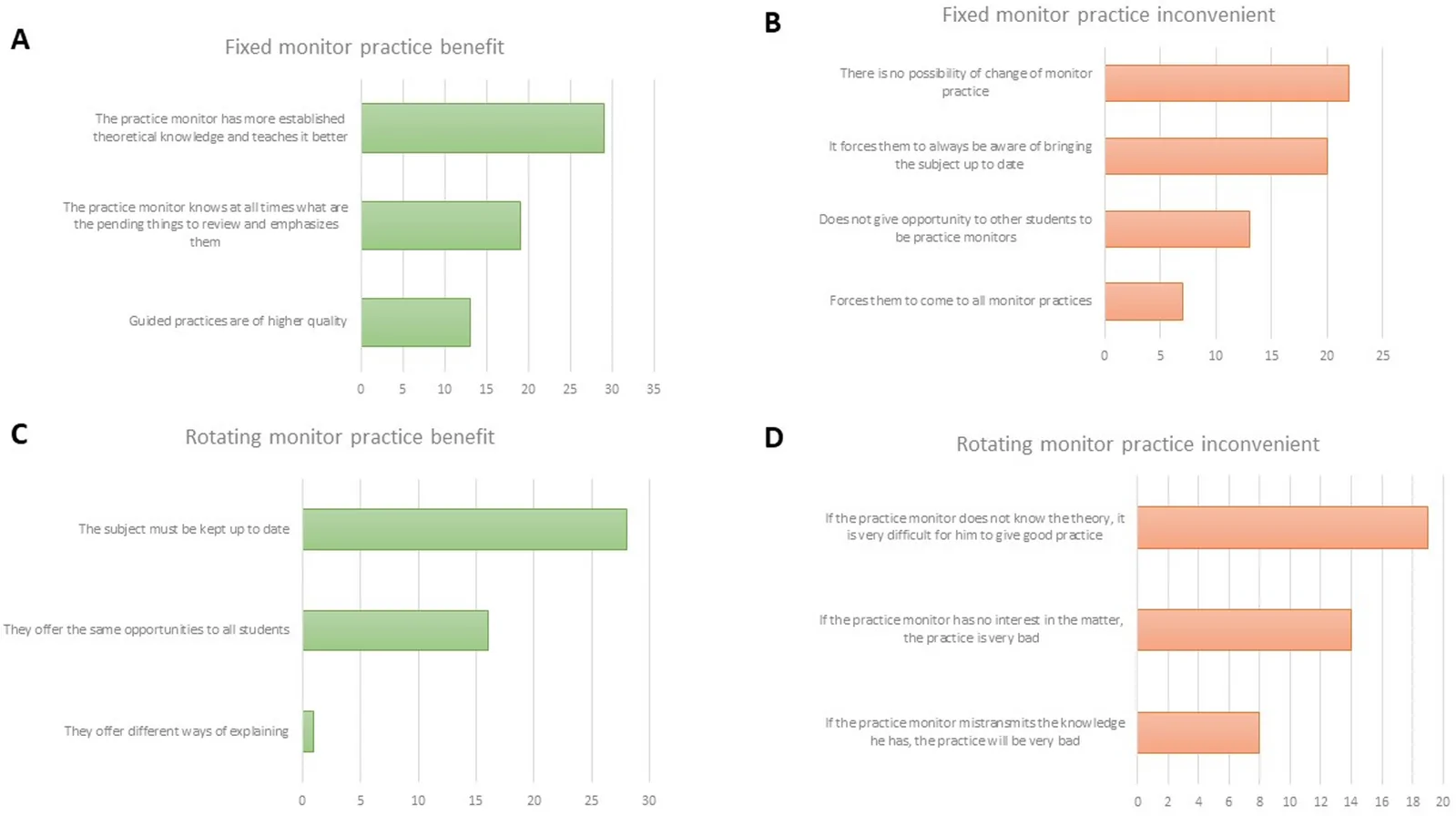
Results about the perception survey about the system of practices in Medical students. (Fig. 3**A**) indicate benefits of fixed practice monitors. (Fig. 3**B**) indicate drawbacks of fixed practice monitors. (Fig. 3**C**) indicate benefits of rotating practice monitors and (Fig. 3**D**) indicate drawbacks rotating practice monitors

The perception of the present study experience was collected from 50.1% (100 out of 198) of all Medical students enrolled in Neuroanatomy subject during the 2022-23 academic year (Fig. 2). Using Likert scales, the students responded favourably to both types of practice monitors, citing their ability to effectively teach the practical lessons and foster teamwork. Of the 100 students who completed the survey, 61% had fixed practice monitors, while 39% had rotating practice monitors. It is interesting to highlight that the type of practice monitors a student had, appeared to influence their preference: 86.8% of students with fixed practice monitors preferred them, and 84.6% of students with rotating practice monitors preferred them. Students from group A indicated that the main benefits of fixed practice monitor were the monitor’s mastery of the lessons and their ability to teach them effectively, as well as the monitor’s constant awareness of pending tasks. Among the biggest drawbacks of fixed practice monitors, students indicated the lack of opportunity to change monitors if they disliked the teaching style. Furthermore, were required to keep their anatomy lessons updated, which could be very time-consuming or detrimental to their other subjects. Regarding rotating practice monitors, students noted that this system requires the monitor to bring the subject up to date and provides equal opportunities to all students. However, they also indicated that if the monitor lacked theoretical knowledge or interest, the quality of the practice session would be extremely low the quality of the practice will be extremely low (Fig. 2).

Finally, the survey included a couple of questions about the fixed practice system, revealing that 68.8% of the students were very happy with their assigned fixed practice monitor and did not want a change. Remarkable, this same 68.8% of students did not wish to be selected for the monitoring role themselves. Conversely, 27.1% would have liked the opportunity to serve as fixed practice monitor. Similar results were found among the elected practice monitor, with 58.3% indicating they enjoyed the role and would like to repeat it, while 29.2% would have preferred not to have been elected.

**Fig. 3:**
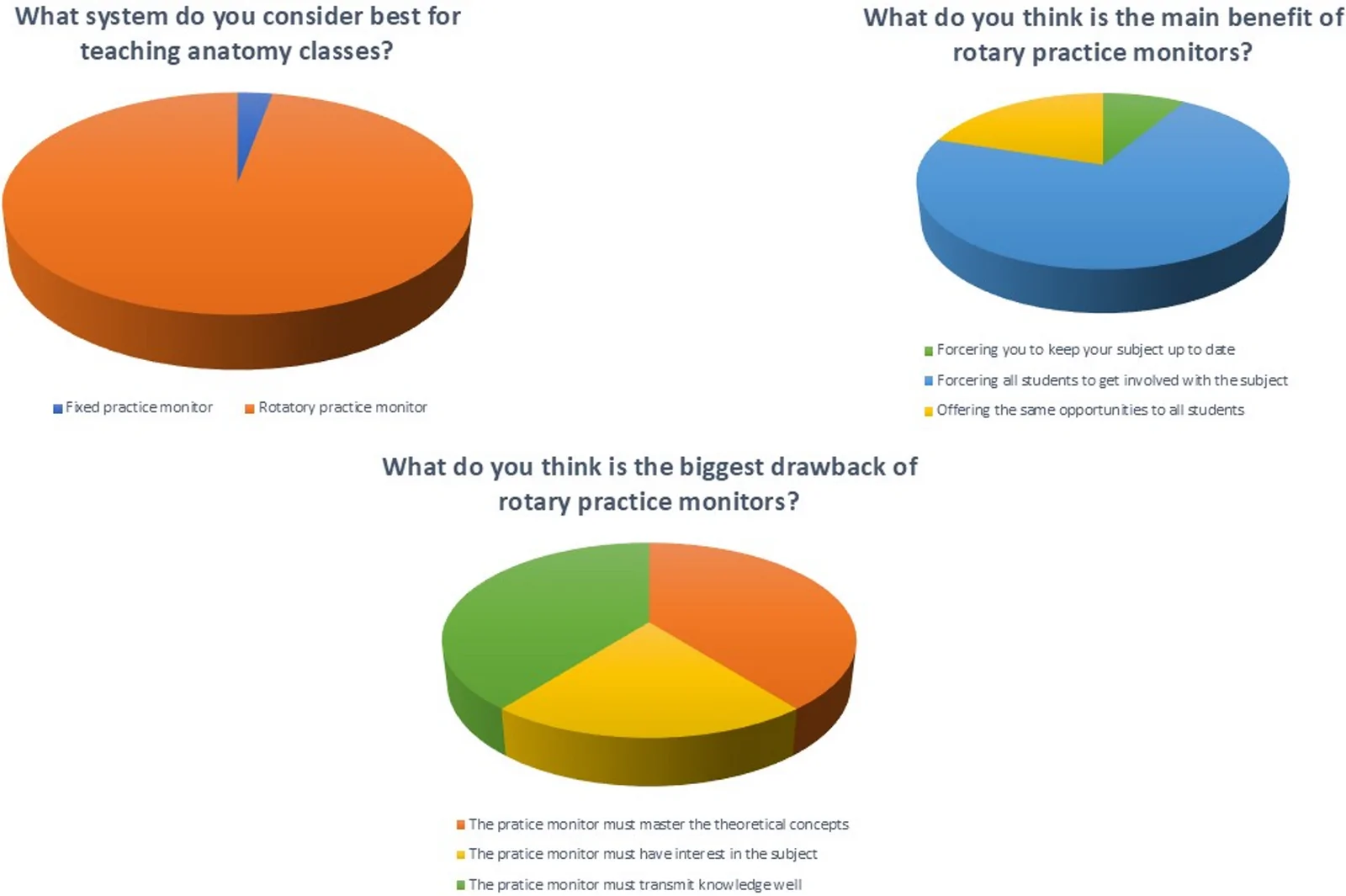
Results about the perception survey about the system of practices in Optometry students

The perception of the present study experience was collected from 60.3% (35 out of 58) of all Optometry students enrolled in Human Anatomy and Histology subject during the 2023-24 academic year (Fig. 3). The majority of these students (97.1%) indicated that their preferred practice system is one with a rotating practice monitor, as they believe it ensures all students are actively engaged in the subject matter. Although they acknowledged potential drawbacks of this system, they highlighted that the practice monitor must possess strong theoretical knowledge, demonstrate an interest in the subject, and be skilled in effectively conveying the knowledge.

## Discusion

Active learning methodologies that place students at the center of the educational process are strongly encouraged within the framework of the European Higher Education Area. While lectures remain an essential component of anatomy education for the structured transmission of information [20, 21], a growing body of evidence supports the added value of active learning strategies in enhancing student engagement, comprehension, and long-term knowledge retention [22, 23]. in this context, small-group practical sessions represent a key element in consolidating theoretical knowledge and are consistently reported as a preferred learning modality by students [18].

Previous studies have highlighted the effectiveness of small-group learning and peer-assisted teaching in anatomy education. For example, Karamaroudis et al. demonstrated that peer teaching was positively received by students and contributed significantly to their understanding of anatomical concepts [24]. Similarly, López et al. reported that collaborative small-group practical sessions using natural specimens, anatomical models, and schematic reconstructions were highly effective in promoting learning and long-term retention in head and neck anatomy courses [19]. These findings support the relevance of structured practical sessions as a complement to traditional lectures.

Neuroanatomy poses particular educational challenges due to the abstract nature of its concepts and the limited availability of tangible structures, which often contribute to students’ perception of the subject as difficult and to the development of “neurophobia” [25–27]. Consequently, considerable effort has been devoted to identifying teaching strategies that improve student engagement and understanding in neuroanatomy, including flipped classrooms, peer teaching, and problem-based learning approaches [28–30]. Flipped classroom and letting the student become the teacher are some of the tips for teaching neuroanatomy from the medical students’ perspective [31].

In line with these approaches, both medical and optometry programs in our institution employ active learning strategies based on small-group practical sessions, consistent with practices reported in other Spanish medical schools [17]. Unlike other models described in the literature [17, 19], each small group in our courses is led by a student monitor who receives targeted preparatory instruction prior to the session and is responsible for guiding peer learning, with faculty available to resolve questions as needed. Within this educational framework, the present study specifically examined whether the organizational model of practice monitor assignment—fixed versus rotating—had an impact on knowledge acquisition and student perceptions. Our findings indicate that, although students assigned to fixed practice monitors demonstrated higher performance in the initial formative assessments, no significant differences were observed between fixed and rotating monitor systems in final quiz performance or final examination results. These results suggest that while continuity in peer leadership may facilitate more consistent early progress, overall learning outcomes are comparable between organizational models. From a curricular perspective, this finding is particularly relevant, as it indicates that flexibility in the allocation of practice monitors can be implemented without negatively affecting academic performance.

Game-based learning strategies, including the use of Kahoot!, have been widely validated as effective tools for formative assessment and student engagement [32–35]. In addition to their motivational value, Kahoot! based activities provide immediate feedback to both students and instructors and facilitate monitoring of learning progress. Although many studies have focused on qualitative outcomes, our previous work demonstrated that performance in Kahoot! activities is predictive of examination results [36]. In the present study, the higher scores observed in early Kahoot! activities among students with fixed practice monitors may reflect greater initial coherence in information delivery. However, as the course progressed, these differences diminished, suggesting that final learning outcomes are influenced predominantly by cumulative self-study and exam preparation rather than by the specific organizational structure of peer-led practical sessions. The absence of significant differences in final outcomes may also be related to the relatively homogeneous academic profile of medical students, resulting from high admission requirements. It is plausible that in more heterogeneous student populations, differences between organizational teaching models might exert a greater influence on learning outcomes. Future studies conducted in diverse educational contexts could help clarify this possibility.

Several limitations of this study should be acknowledged. First, Kahoot! activities were completed at the group level, which may have led to repeated participation by the same students, potentially overrepresenting higher-performing individuals. Second, medical students were exposed to only one practice monitor system, limiting direct within-group comparisons of student perceptions. Although a crossover design would have strengthened this aspect, logistical constraints and the single-semester structure of the neuroanatomy course precluded its implementation. Finally, comparisons between different student groups may be influenced by baseline differences in academic ability. This limitation was partially mitigated by confirming the absence of significant differences in prior splanchnology grades between groups and by the random assignment of students to groups, which remained consistent throughout the medical program. In optometry students, comparisons were performed within the same cohort, further strengthening the internal validity of these findings.

## Conclusion

This study evaluated the impact of two organizational models for small-group practical sessions—fixed and rotating practice monitors—on knowledge acquisition and academic performance in Anatomy courses within Medicine and Optometry programs. Our findings indicate that students achieved comparable final examination results regardless of the practice monitor system implemented. However, differences were observed in early formative assessments, suggesting that fixed practice monitor systems may support more consistent and progressive knowledge acquisition during the initial stages of the course. Overall, both organizational models appear to be educationally valid, allowing flexibility in the assignment of practice monitors without compromising final learning outcomes.

## Supplementary Information

Below is the link to the electronic supplementary material.

Supplementary Material 1

Supplementary Material 2

Supplementary Material 3
