# Supplementary material for: Fixed Versus Rotating Practice Monitors in Small-Group Anatomy Teaching: A Prospective Study in Medicine and Optometry
Source: Med Sci Educ. 2026 Jun 29;36(4):1809–17. doi: 10.1007/s40670-026-02793-6 (PMC13582690; doi:10.1007/s40670-026-02793-6)
Supplement: Supplementary file 1 [file 40670_2026_2793_MOESM2_ESM.docx]

**Supplementary Table 1.**  Theoretical and practical classes in the Neuroanatomy subject of the Medicine degree.

| **Type of class** | **Title** |
| --- | --- |
| Theoretical | Unit 1. Introduction to the Nervous System |
| Theoretical | Unit 2.- Sense organs: Touch, taste and smell |
| Theoretical | Unit 3.-Sense of Hearing: Inner ear, Middle ear, Outer ear |
| Theoretical | Unit 4.- Sense of Sight. Eyeball, walls: Retina, Vascular layer, Fibrous layer |
| Theoretical | Unit 5.- Sense of sight. Eyeball, content: Lens, Humors |
| Theoretical | Unit 6.- Oculomotor neuromuscular systems |
| Theoretical | Unit 7.- Orbital content and protection organs |
| Theoretical | Unit 8.- Configuration, structure, vascularization and envelopes of the spinal cord |
| Theoretical | Unit 9.- Medullary sensory systems |
| Theoretical | Unit 10.- Vegetative effectors in the medulla |
| Theoretical | Unit 11.- Somatic motor effectors in the medulla. Spinal cord reflexes |
| Theoretical | Unit 12.- Configuration, structure and vascularization of the brainstem |
| Theoretical | Unit 13.- Brainstem sensory systems |
| Theoretical | Unit 14.- Vegetative effectors in the brainstem |
| Theoretical | Unit 15.- Somatic motor effectors in the brainstem |
| Theoretical | Unit 16.- Cranial nerves |
| Theoretical | Unit 17.- Reticular formation |
| Theoretical | Unit 18.- Colliculi and Substantia Nigra |
| Theoretical | Unit 19.- Brainstem nuclei related to the cerebellum |
| Theoretical | Unit 20.- Configuration, vascularization and envelopes of the cerebellum |
| Theoretical | Unit 21.- Structure of the cerebellum |
| Theoretical | Unit 22.- Archicerebellum/Vestibulo-Cerebellum |
| Theoretical | Unit 23.- Paleocerebellum/Spino-Cerebellum, Neocerebellum/Cerebro-Cerebellum |
| Theoretical | Unit 24. - Configuration, structure and vascularization of the diencephalon. circumventricular organs |
| Theoretical | Unit 25.- Hypothalamus. Hypophysis, |
| Theoretical | Unit 26.- Epithalamus. Pineal gland |
| Theoretical | Unit 27.- Thalamus |
| Theoretical | Unit 28.- Subthalamus. Telencephalic basal nuclei |
| Theoretical | Unit 29.- Internal configuration of the telencephalon. white matter |
| Theoretical | Unit 30.- Internal configuration of the telencephalon. Lateral ventricles. |
| Theoretical | Unit 31.- External configuration of the cerebral cortex. |
| Theoretical | Unit 32.- Structure of the cerebral cortex |
| Theoretical | Unit 33.- Sensory cortices: somatoesthetic, acoustic and visual |
| Theoretical | Unit 34.- Gustatory and olfactory cortex |
| Theoretical | Unit 35.- Hippocampus and limbic system |
| Theoretical | Unit 36.- Association cortices and frontal lobe |
| Theoretical | Unit 37.- Motor cortices |
| Theoretical | Unit 38.- Arterial vascularization of the brain |
| Theoretical | Unit 39.- Venous return of the brain |
| Theoretical | Unit 40.- Meninges and CSF circulation. brain barriers |
| Theoretical | Unit 41.- Sensory pathways |
| Theoretical | Unit 42.- Somato-motor pathways |
| Theoretical | Unit 43.- Viscero-motor pathways |
| Theoretical | Unit 44.- Radiological anatomy and image study techniques of the Nervous System |
| Practical | Study of the oral and nasal cavities: taste and smell |
| Practical | Study of the organs of hearing and balance. Boulder |
| Practical | Study of the organs of vision. orbital cavity |
| Practical | Macroscopic study of the marrow |
| Practical | Study of medullary sections |
| Practical | Macroscopic study of the brainstem |
| Practical | Study of brainstem cuts and reconstructions |
| Practical | Macroscopic study of the cerebellum |
| Practical | Study of cuts and reconstructions of the cerebellum |
| Practical | Macroscopic study of the diencephalon |
| Practical | Study of cuts and reconstructions of the diencephalon |
| Practical | External and internal macroscopic study of the telencephalon |
| Practical | Study of sections and reconstructions of the telencephalon |
| Practical | Study with imaging techniques of the Nervous System |

**Supplementary Table 2.**  Titles of Theoretical and practice class in the Human Anatomy and Histology subject imparted in the Optometry Grade.

| **Type of class** | **Title** |
| --- | --- |
| Theoretical | Unit 1. Concept of morphology. Cell. |
| Theoretical | Unit 2. Embryology: formation of blastodermal layers. |
| Theoretical | Unit 3. Tissues. Classification |
| Theoretical | Unit 4. Epithelial tissue. |
| Theoretical | Unit 5. Connective tissue. |
| Theoretical | Unit 6. Adipose tissue. |
| Theoretical | Unit 7. Cartilage tissue. |
| Theoretical | Unit 8. Bone tissue. |
| Theoretical | Unit 9. Muscle tissue. |
| Theoretical | Unit 10. Nervous tissue I. |
| Theoretical | Unit 11. Nervous tissue II. |
| Theoretical | Unit 12. Blood. Immune system. |
| Theoretical | Unit 13. Organogenesis |
| Theoretical | Unit 14. Embryology of the Head I. Development of the Central Nervous System. |
| Theoretical | Unit 15. Embryology of the Head II. Development of the Head as a Whole |
| Theoretical | Unit 16. Craniofacial Development |
| Theoretical | Unit 17. Development of the Sense Organs |
| Theoretical | Unit 18. Development of the Visual Apparatus |
| Theoretical | Unit 19. Introduction to Anatomy, Planes, and Axes |
| Theoretical | Unit 20. Circulatory System I. Heart |
| Theoretical | Unit 21. Circulatory System II |
| Theoretical | Unit 22. Respiratory System |
| Theoretical | Unit 23. Digestive System |
| Theoretical | Unit 24. Locomotor System I. Neck, Spine, Lower Extremity |
| Theoretical | Unit 25. Locomotor System II |
| Theoretical | Unit 26. Endocrine System |
| Theoretical | Unit 27. Urinary System |
| Theoretical | Unit 28. Male Genital System |
| Theoretical | Unit 29. Female Genital System |
| Theoretical | Unit 30. Integumentary System |
| Theoretical | Unit 31. Skull Base |
| Theoretical | Unit 32. Cranial Vault |
| Theoretical | Unit 33. Viscerocranium |
| Theoretical | Unit 34. Orbital Fossa |
| Theoretical | Unit 35. Introduction to the Sense of Sight |
| Theoretical | Unit 36. Anatomy of the Eyeball: Chambers |
| Theoretical | Unit 37. Internal Tunic. Retina I |
| Theoretical | Unit 38. Internal Tunic. Retina II |
| Theoretical | Unit 39. Median Tunic (Uvea): Choroid |
| Theoretical | Unit 40. Median Tunic, Ciliary Body |
| Theoretical | Unit 41. Median Tunic, Iris |
| Theoretical | Unit 42. External Tunic: Sclera, Cornea |
| Theoretical | Unit 43. External Tunic: Sclera, Cornea. Sclerocorneal Angle |
| Theoretical | Unit 44. Refractive Media: Lens |
| Theoretical | Unit 45. Refractive Media: Aqueous Humor. Vitreous Body |
| Theoretical | Unit 46. External Vision of the Eyeball: Eyebrows |
| Theoretical | Unit 47. Eyelids and Conjunctiva |
| Theoretical | Unit 48. Lacrimal system (gland and ducts). |
| Theoretical | Unit 49. Extrinsic musculature. |
| Theoretical | Unit 50. Optic nerve. |
| Theoretical | Unit 51. Sensory innervation. |
| Theoretical | Unit 52. Autonomic innervation of the visual apparatus and related organs. |
| Theoretical | Unit 53. Arterial supply of the orbital fossa. |
| Theoretical | Unit 54. Venous drainage of the orbital fossa. |
| Theoretical | Unit 55. Contents of the orbital fossa. |
| Theoretical | Unit 56. Surface anatomy of the visual sense. |
| Theoretical | Unit 57. Anatomy of the central nervous system. Spinal cord. Metameric reflex. |
| Theoretical | Unit 58. CNS Anatomy. Spinal Cord. Ascending and Descending Tracts. |
| Theoretical | Unit 59. CNS Anatomy. Brainstem. Motor and Sensory Nuclei |
| Theoretical | Unit 60. CNS Anatomy. Brainstem. Reticular Formation |
| Theoretical | Unit 61. CNS Anatomy. Cerebellum |
| Theoretical | Unit 62. CNS Anatomy. Diencephalon. Thalamus and Subthalamus |
| Theoretical | Unit 63. CNS Anatomy. Diencephalon. Hypothalamus, Pituitary Gland, Epithalamus, Epiphysis |
| Theoretical | Unit 64. CNS Anatomy. Telencephalon. |
| Theoretical | Unit 65. CNS Anatomy. Meninges, CSF, and Cerebral Irrigation. |
| Theoretical | Unit 66. Visual Pathway I |
| Theoretical | Unit 67. Visual Pathway II |
| Practical | Use of the Optical Microscope |
| Practical | Recognition of different histological tissues using an optical microscope |
| Practical | Recognition of the most important organs of the human body and the components of the sense of sight during embryonic development using models and atlases |
| Practical | Dissection of the heart |
| Practical | Skeleton of the skull |
| Practical | Skeleton of the orbital fossa |
| Practical | Recognition of the components of the eyeball using atlases and models |
| Practical | Recognition of the contents of the orbital fossa using models and atlases |
| Practical | Dissection of the eyeball |
| Practical | Dissection of the orbit |
| Practical | Recognition of the main components of the CNS using models and atlases |
| Practical | Recognition of the components of the optic pathway using models and atlases |
