# Supplementary material for: Fixed Versus Rotating Practice Monitors in Small-Group Anatomy Teaching: A Prospective Study in Medicine and Optometry
Source: Med Sci Educ. 2026 Jun 29;36(4):1809–17. doi: 10.1007/s40670-026-02793-6 (PMC13582690; doi:10.1007/s40670-026-02793-6)
Supplement: Supplementary file 2 [file 40670_2026_2793_MOESM3_ESM.docx]

4^th^ June 2026

**Dear Editor-in-Chief,**

Prof. David M. Harris, PhD

Please find enclosed the revised version of our manuscript entitled “Analyzing a peer teaching methodology in anatomy: fixed versus rotating practice monitors”, which we resubmit as an Original Research article for consideration in Medical Science Educator.

We would like to thank the Editor and the reviewers for their thoughtful and constructive comments. We have carefully revised the manuscript in response to all reviewer suggestions, with particular attention to improving the clarity of the language, strengthening the accuracy of the references in the Introduction, and enhancing the overall presentation of the Methods, Results, and Discussion sections.

All changes requested by the reviewers have been thoroughly addressed, and all modifications in the revised manuscript have been highlighted in yellow to facilitate review.

We respectfully ask that the revised manuscript be considered again for possible publication in Medical Science Educator.

On behalf of all authors, I confirm that the manuscript is original, has not been published previously, and is not under consideration elsewhere. All authors have reviewed and approved the revised version.

Thank you for your time and consideration.

Sincerely,
Itziar Lamiquiz-Moneo, PhD

Department of Human Anatomy and Histology, School of Medicine, University of Zaragoza
